# Supplementary material for: Electrosynthesis of ethylene glycol from C1 feedstocks in a flow electrolyzer
Source: Nat Commun. 2023 Jul 29;14:4570. doi: 10.1038/s41467-023-40296-9 (PMC10387065; doi:10.1038/s41467-023-40296-9)
Supplement: Supplementary file 1 — Supplementary Information [file 41467_2023_40296_MOESM1_ESM.pdf]

Supporting Information

Electrosynthesis of Ethylene Glycol from C_1 feedstocks through C-C Coupling

Rong Xia[ab], Ruoyu Wang[c], Bjorn Hasa[b], Ahryeon Lee[b], Yuanyue Liu[c]*, Xinbin Ma[a]*, Feng Jiao[b]*

Affiliations:
[a]Key Laboratory for Green Chemical Technology, School of Chemical Engineering and Technology, Tianjin University, Tianjin 300072, China
[b]Center for Catalytic Science and Technology, Department of Chemical and Biomolecular Engineering, University of Delaware, Newark, DE 19716, United States
[c]Texas Materials Institute and Department of Mechanical Engineering, The University of Texas at Austin, Austin, TX, 78712, USA

*Corresponding authors: jiao@udel.edu, xbma@tju.edu.cn, yuanyue.liu@austin.utexas.edu

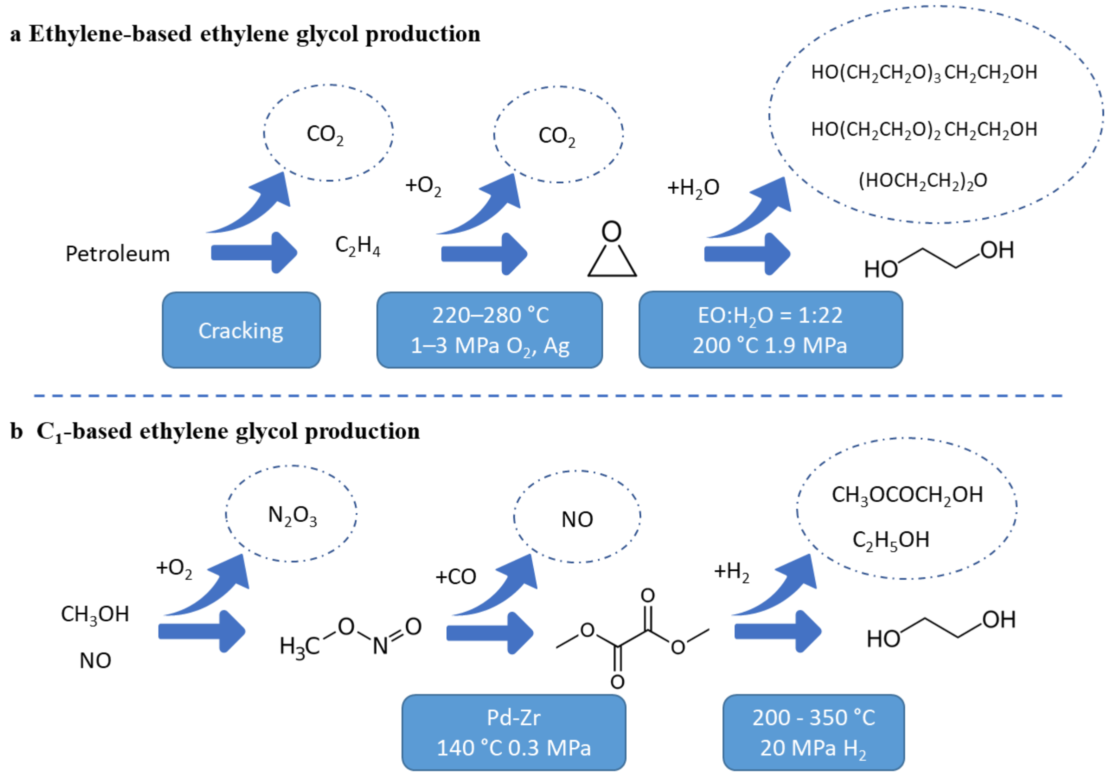

Figure S 1| Schematics of convectional thermocatalytic routes for ethylene glycol production. a) Ethylene-based ethylene glycol production. b) C_1-based ethylene glycol production.

Table S 1| Bulk price of methanol, formalin (37% formaldehdye solution), ethylene glycol and ethylene.

Feedstocks	Methanol	Formaldehyde 37 wt.%	Ethylene	Ethylene glycol
Bulk price USD/ton	350[1]	450[2]	1235[3]	838[4]

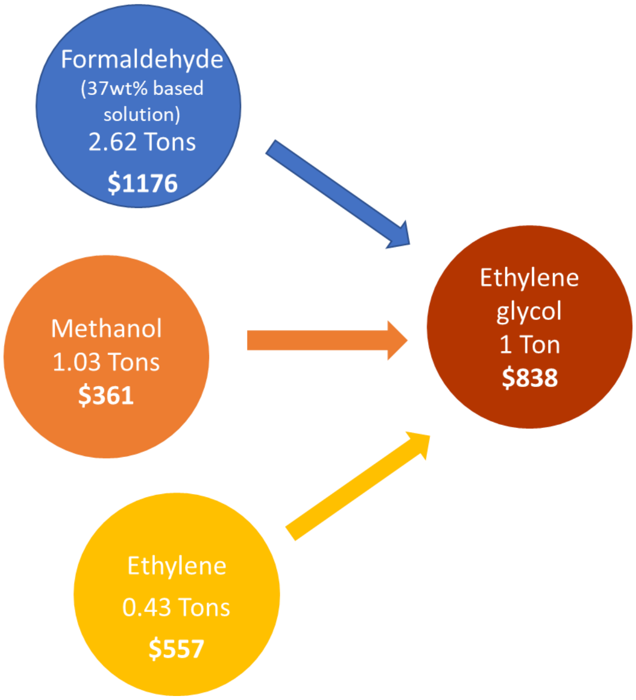

Figure S 2| The price of feedstock to make 1 ton of ethylene glycol from methanol, formaldehyde, and ethylene, respectively.

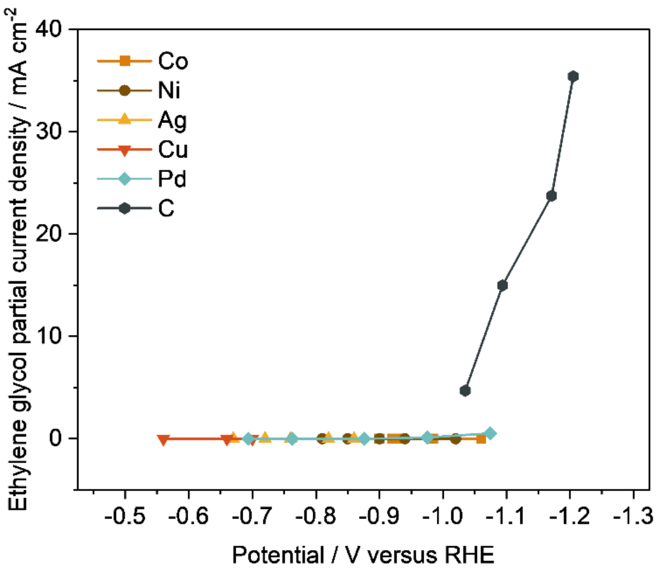

Figure S 3| Ethylene glycol partial current density in formaldehdye electroreduction on Co, Ni, Ag, Cu, Pd and C, respectively. The experiment was performed in 37 wt% formaldehyde solution containing 1 M sodium acetate as supporting electrolyte under ambient temperature and pressure.

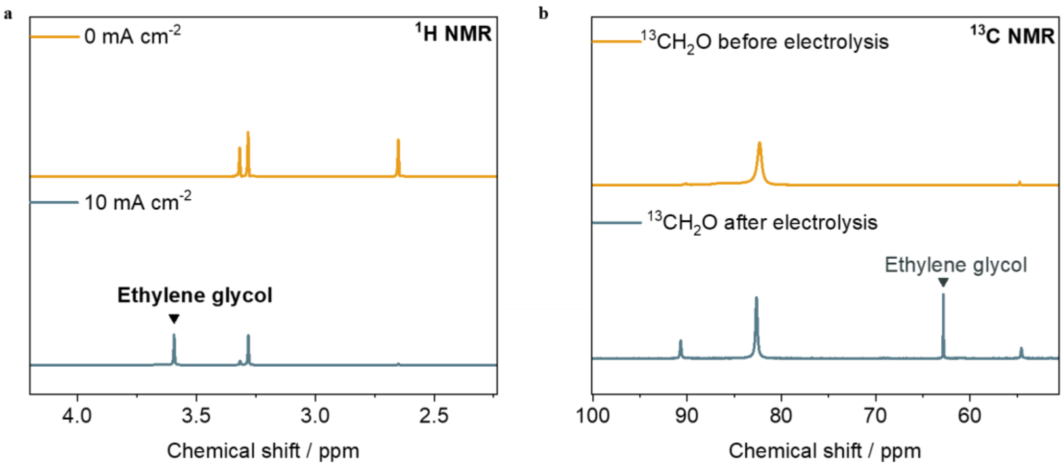

Figure S 4| ^{1}H NMR spectra (a) and ^{13}C NMR spectra of product generated in formaldehyde reduction on carbon catalysts using 4 wt% carbon-13-labeled formaldehyde ($^{13}CH_2O$) as starting material.

The carbon source of ethylene glycol is examined using nuclear magnetic resonance (NMR) and gas chromatography–mass spectrometry (GC-MS). The ^{13}C-labeled formaldehyde ($^{13}CH_2O$) is used as starting material, and formaldehyde electroreduction is conducted at a constant current of 10 mA cm^{-2} for 10 hours using carbon black as catalysts. The ^{13}C-labeled ethylene glycol is identified as the product in the ^{13}C and ^{1}H NMR spectra (Figure S4). To investigate whether formaldehyde is the sole carbon source, the resulting ethylene glycol was analyzed by GC-MS. The ethylene glycol synthesized using $^{13}CH_2O$ feedstocks exhibited an apparent 2 amu mass shift in ethylene glycol compared with that generated from non-labeled formaldehyde (Figure 2b), suggesting that both carbon atoms were originated from $^{13}CH_2O$. The trace signals at 63 and 64 amu in ethylene glycol from non-labeled formaldehyde are due to the natural abundance of ^{13}C in $^{12}CH_2O$ solution. A controlled experiment was performed without formaldehyde using the same carbon catalyst. Only hydrogen is detected, and no ethylene glycol is observed in ^{1}H NMR spectra, confirming that neither carbon catalysts nor supporting electrolyte contribute to the formation of ethylene glycol.

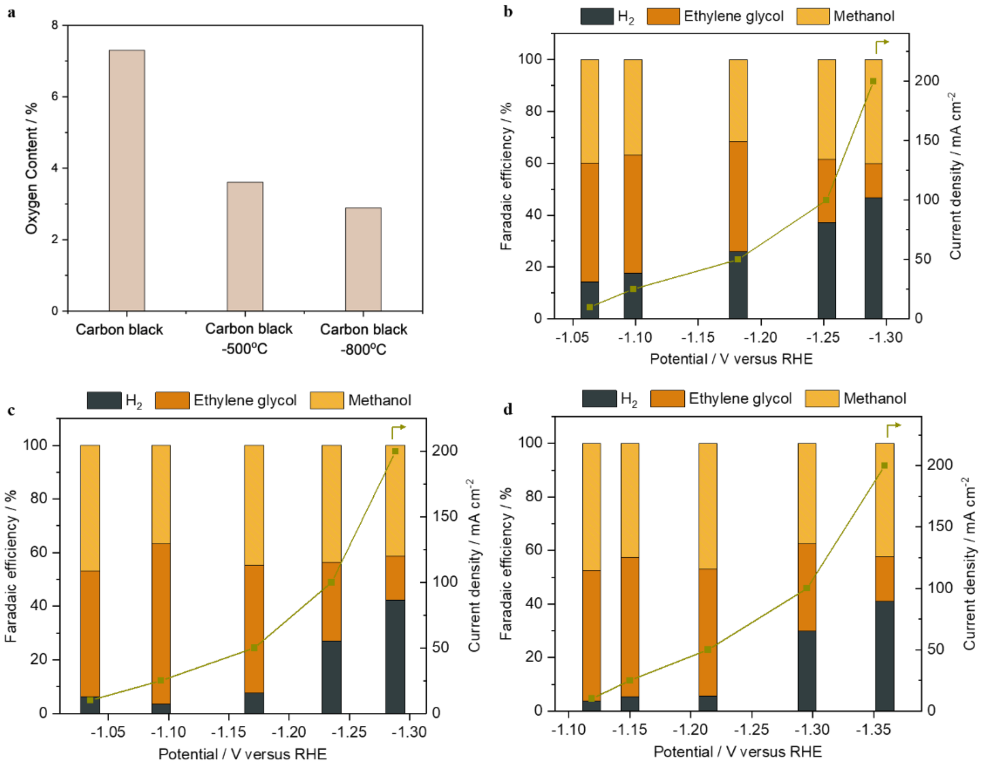

Figure S 5| Thermal treatment effects on the removal of oxygen-containing functional groups on carbon black catalysts and the subsequent formaldehyde electroreduction performance. (a) The oxygen content of carbon black catalysts after undergoing thermal treatment at 500°C and 800°C, respectively. Comparative performance of formaldehyde electroreduction using carbon black catalysts without thermal treatment (b), and those treated at 500°C (c) and 800°C (d).

The oxygen-containing functional group in carbon black catalysts can be further reduced by thermal treatment at elevated temperatures. The relationship between temperature and oxygen content is depicted in Figure S5. When subjected to a calcination process at 500°C under a 5% H_2/Ar atmosphere, the oxygen content in carbon black falls from 7.3% to 3.6%. As the thermal treatment temperature rises from 500°C to 800°C, the oxygen content further reduces to 2.9%. We tested carbon black catalysts, with varying oxygen contents, for formaldehyde electroreduction under identical conditions. Our results indicate that by eliminating oxygen functional groups via thermal treatment, the Faradaic efficiency (FE) for ethylene glycol improved, and side reactions were minimized. However, extended annealing at higher temperatures was unable to remove additional oxygen functional groups and led to a degradation in the pore properties and surface area of the carbon black, as documented in earlier research.[5] A similar phenomenon was also noticed when using commercial graphene. Thermal treatment was unable to fully eliminate the oxygen-containing functional groups on the graphene, resulting in a residual oxygen content of 2.3% post-heat treatment. This can be attributed to the fact that the edge surface of graphene tends to remain completely oxygenated, as stated in previous literature.[6]

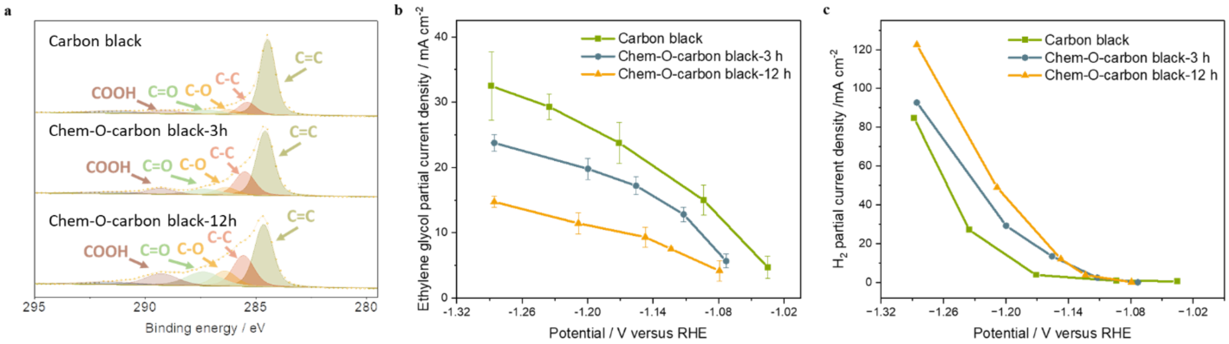

Figure S 6 | Carbon black with various oxygen content and its performance in formaldehyde electroreduction. a) C 1s X-ray photoelectron spectroscopy of carbon black treated in 70 wt% nitric acid at 70°C for 3 hours and 12 hours, denoted as Chem-O-carbon black-3 h and Chem-O-carbon black-12 h. b) Ethylene glycol partial current density on carbon black with different oxygen content. c) Hydrogen partial current density on carbon black with different oxygen content. Error bars represent the standard deviation in three independent measurements.

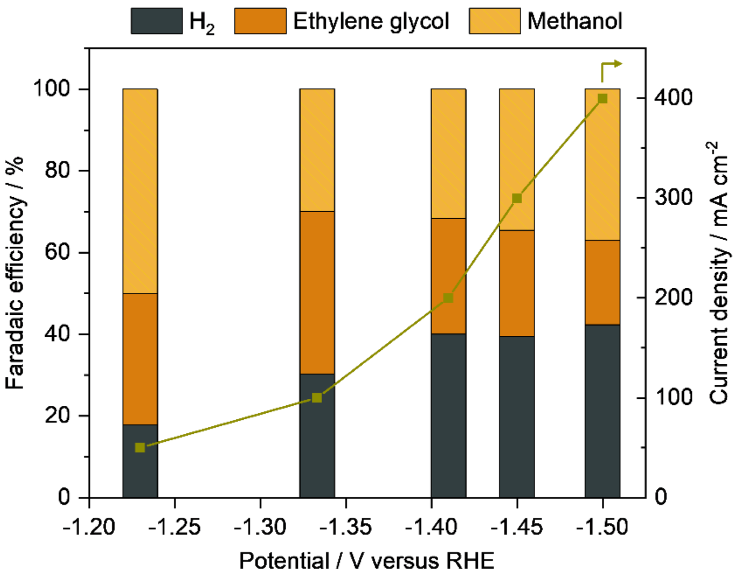

Figure S 7| Formaldehyde electroreduction on blank carbo paper. The blank carbon paper is tested for formaldehdye electroreduction and exhibits much higher overpotential and higher hydrogen FE. This can be attributed to the high loading of PTFE in carbon paper covered the active sites of carbon.

Supplementary note: Local concentration of free formaldehyde. The schematic of the electrocatalytic reaction system is shown in Figure S8. As it can be seen, the reaction system is divided by into the catalyst layer and bulk solution layer. For both layers, free formaldehyde participated in a reversible hydrolysis reaction to $CH_2(OH)_2$ as shown in Eq.(1) and Eq.(2). The rate of reaction expression can be described in a simple elementary rate expression shown in Eq.(3) and Eq.(4), respectively. The rate constants were obtained from literature and shown in Eq.(5) and Eq.(6).[7] Combing Eq.(3) through Eq.(6), the equilibrium concentration of free formaldehyde in 37 wt% formaldehyde solution was computed. It is found that higher temperatures result in more free formaldehyde concentrations but its level (0.0005 – 0.0015 M) is significantly lower than the $CH_2(OH)_2$ concentrations (11.4 M). In this modeling work, it is assumed that the free formaldehyde is only consumed by electrocatalytic reaction (Eq.(7) within 1μm of carbon catalyst layer thickness with a porosity (ε) of 50% due to nature of heterogeneous reaction. Meanwhile, the competing HER reaction also occurred only in the 1μm catalyst layer. (Eq.(9).[8] The rate expression for main and side electrocatalytic reaction are shown in Eq.(8) and Eq.(10), respectively. The j is current density, ε is porosity of the electrode, assuming 0.3. F is the faraday constant, and $FE_{C_2H_6O_2}$ and FE_{H_2} are the faraday efficiency of the ethylene glycol and hydrogen gas formation, respectively which are determined experimentally. $n_{e,C_2H_6O_2}$ and $n_{e,\,H_2}$ are the amount of electron transfer which equal to $2e^-$. In Figure 8, for the bulk solutions phase that is beyond 1μm, it is assumed that rate of electrocatalytic formaldehyde conversion is negligible due to nature of heterogeneous reaction, and only reversible hydration/dehydration of formaldehyde occurred. This assumption is adapted from our previous kinetics modelling work that was used for the similar flow cell set up.

$$CH_2O+H_2O \overset{k_h}{\rightarrow} CH_2(OH)_2 \tag{1}$$

$$CH_2(OH)_2 \overset{k_d}{\rightarrow} CH_2O+H_2O \tag{2}$$

$$r_h=k_h C_{CH_2O} C_{H_2O} \tag{3}$$

$$r_d=k_d C_{CH_2(OH)_2} \tag{4}$$

$$K_h=\frac{k_h}{k_d}=\exp(\frac{3769}{T}-5.494) \tag{5}$$

$$k_h=2.04\times10^5\times \exp\left(-\frac{2936}{T}\right) \tag{6}$$

A reaction kinetics model is developed focus only on the catalyst layer, and the mass transfer between catalyst layer and the bulk solution can be described by mass transfer equation in Eq.(11). The mass transfer coefficient of free and dehydrated formaldehyde is 8.12×10^{-5} m/s.[9] The water mass transfer coefficient is. Combining free formaldehyde equilibrium hydrolysis/dehydration reaction, ethylene glycol and hydrogen gas electrosynthesis reaction and mass transfer equation, a system of ordinary differential equations are constructed from Eq.(12) to Eq.(16) which solves the concentration of reaction species change over residence time. Residence time τ is defined in the Eq.(17), which equals to the distance to the flow reactor

inlet *z* over linear velocity of flow *v* in the flow cell. By solving the systems of ordinary differential equation (ODE) equations, the free formaldehyde concentrations and other species concentration can be simulated cross the residence time. The free formaldehyde and hydrated formaldehyde were simulated for 70°C under 0 mA/cm^2 and 200 mA/cm^2 current density. The results show that both free formaldehyde and hydrated formaldehyde remains at equilibrium constant concentration when current density is 0 mA/cm^2. At 200 mA/cm$_2$, both free formaldehyde and hydrated formaldehyde are consumed as flow through the flow cell and remain constant after coming out of flow reactor (beyond 1cm). In both cases, the free formaldehyde is significantly lower than the hydrated formaldehyde due to abundance of water in the presence.

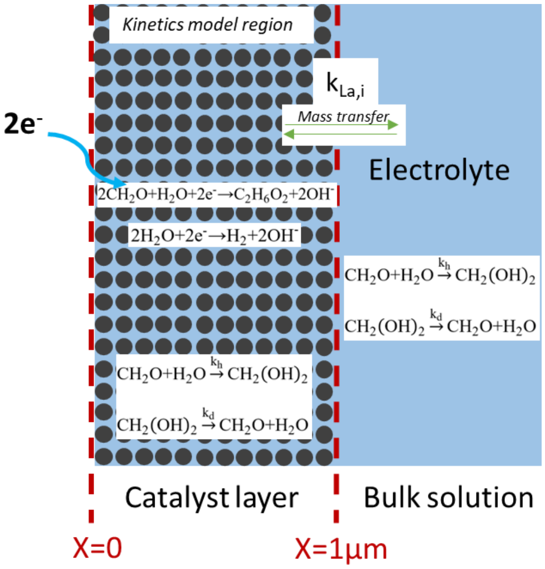

Figure S 8| Schematic of the kinetics model region and model assumptions

$$2CH_2O+H_2O+2e^-\rightarrow C_2H_6O_2+2OH^- \tag{7}$$

$$Rxn_{C_2H_6O_2}=\frac{j\varepsilon}{F\times(1\mu m)}\left(\frac{FE_{C_2H_6O_2}}{n_{e,C_2H_6O_2}}\right) \tag{8}$$

$$2H_2O+2e^-\rightarrow H_2+2OH^- \tag{9}$$

$$Rxn_{H_2}=\frac{j\varepsilon}{F\times(1\mu m)}\left(\frac{FE_{H_2}}{n_{e,\ H_2}}\right) \tag{10}$$

$$\dot{n}_i=-k_{La,i}(c_{RXN,i}-c_{bulk,i}) \tag{11}$$

$$\frac{dCH_2O}{d\tau}=-2Rxn_{C_2H_6O_2}+r_d+\dot{n}_{CH_2O} \tag{12}$$

$$\frac{dCH_2(OH)_2}{d\tau}=r_h-r_d+\dot{n}_{CH_2(OH)_2} \tag{13}$$

$$\frac{dH_2O}{d\tau}=-2Rxn_{H_2}-r_h+r_d+\dot{n}_{H_2O} \tag{14}$$

$$\frac{dC_2H_6O_2}{d\tau}=Rxn_{C_2H_6O_2} \tag{15}$$

$$\frac{dH_2}{d\tau}=Rxn_{H_2} \tag{16}$$

$$\tau=\frac{z}{v} \tag{17}$$

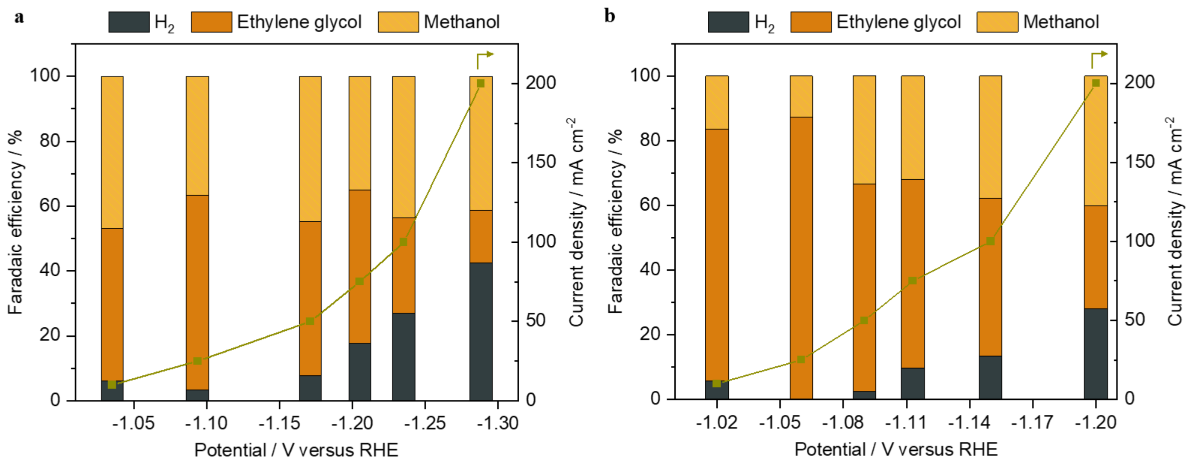

Figure S 9| Performance of formaldehyde electroreduction on carbon under various temperatures. (a) Formaldehyde electroreduction at 30ºC. (b) Formaldehyde electroreduction at 40ºC.

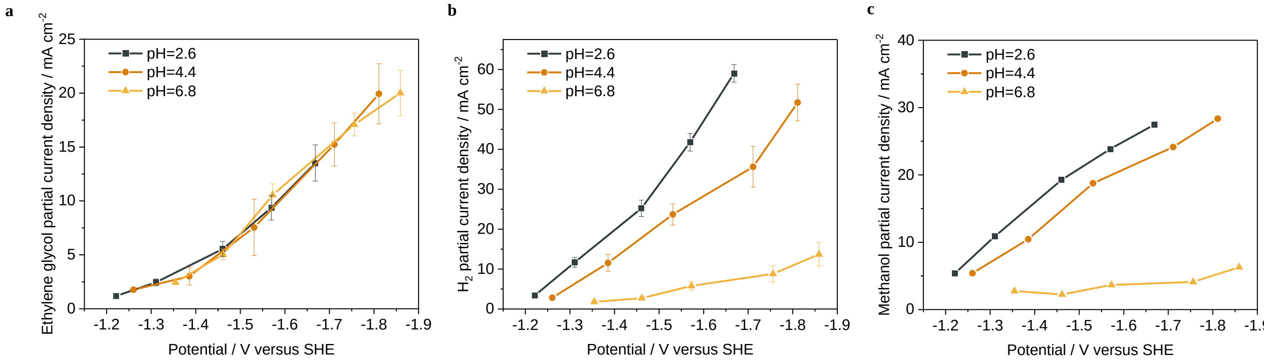

Figure S 10| The pH dependence of ethylene glycol (a), hydrogen (b), methanol (c) formation in formaldehdye electroreduction. Error bars represent the standard deviation in three independent measurements.

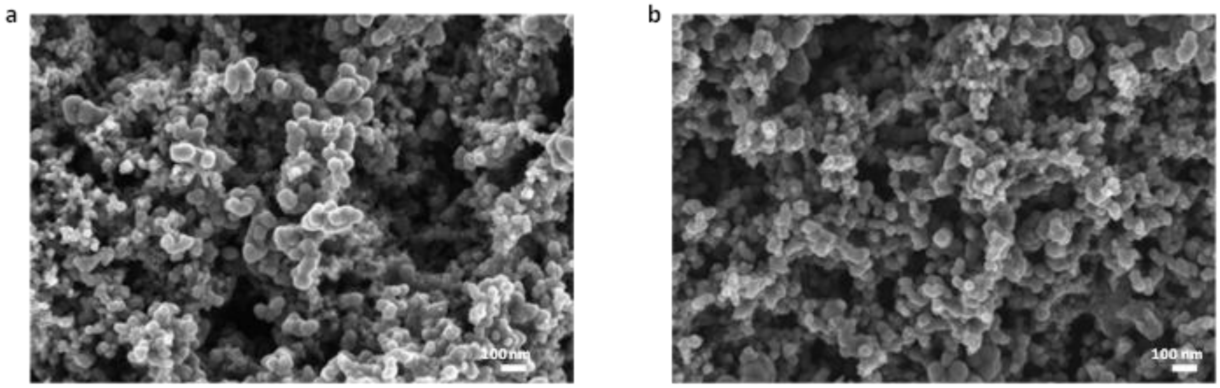

Figure S 11| SEM images of carbon catalyst before (a) and after (b) 10-hour stability test at 100 mA cm^{-2}.

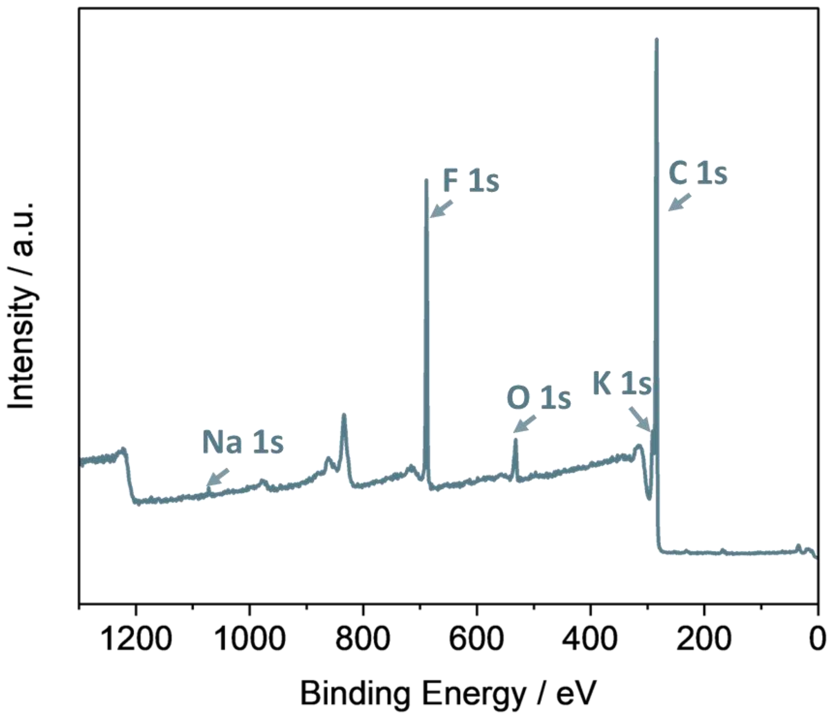

Figure S 12| XPS spectra of post-reaction carbon catalyst after 10-hour stability test. The F signal is from Nafion ionomer used as a binder.

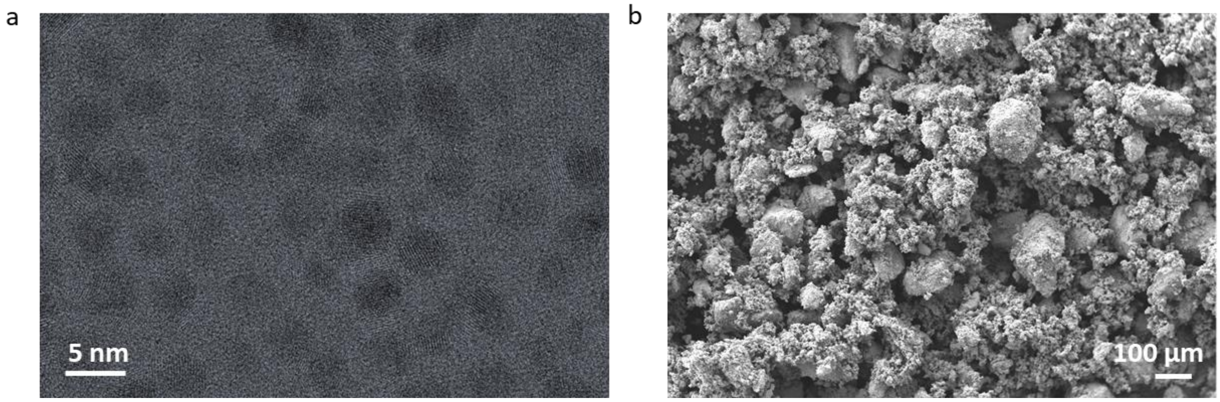

Figure S 13| Morphology of Pt nanoparticles (a) and Pt microparticles (b).

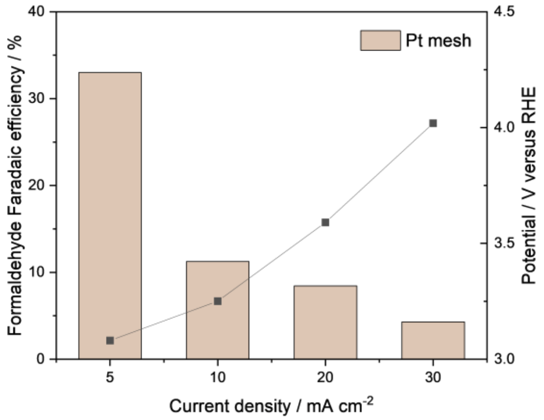

Figure S 14|Performance of methanol partial oxidation to formaldehyde on Pt mesh.

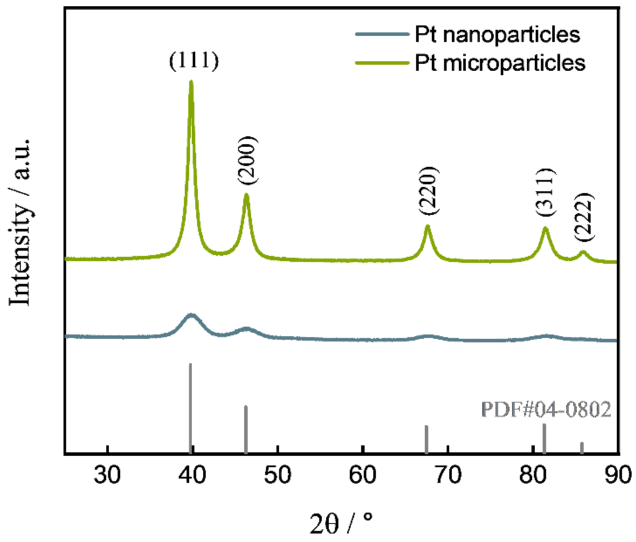

Figure S 15| XRD patterns of Pt nanoparticles and Pt microparticles.

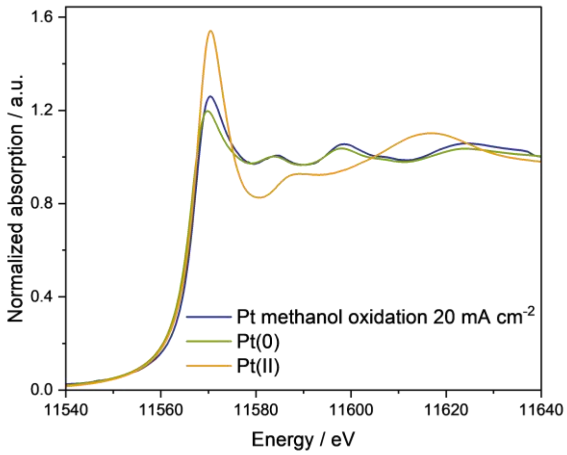

Figure S 16| The X-ray absorption near edge structure (XANES) spectra of Pt microparticles under the methanol electrochemical partial oxidation reaction condition compared with Pt(0) and Pt(II) standard.

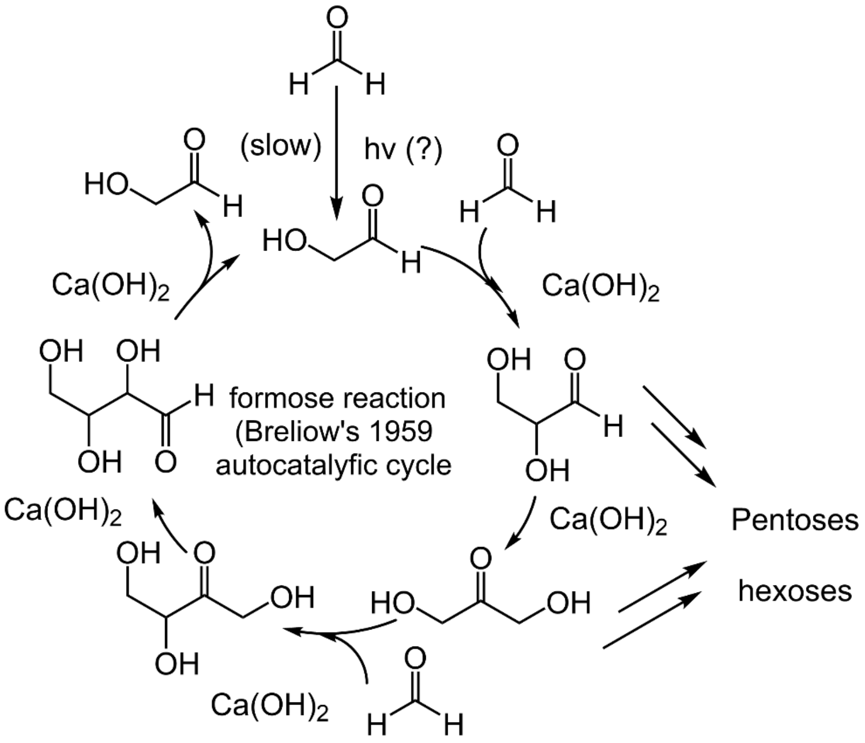

Figure S 17| The reaction mechanism of formose reaction to make glycolaldehyde and higher carbohydrates from formaldehyde, catalyzed by divalent metal cations such as Ca^{2+} and Mg^{2+}.

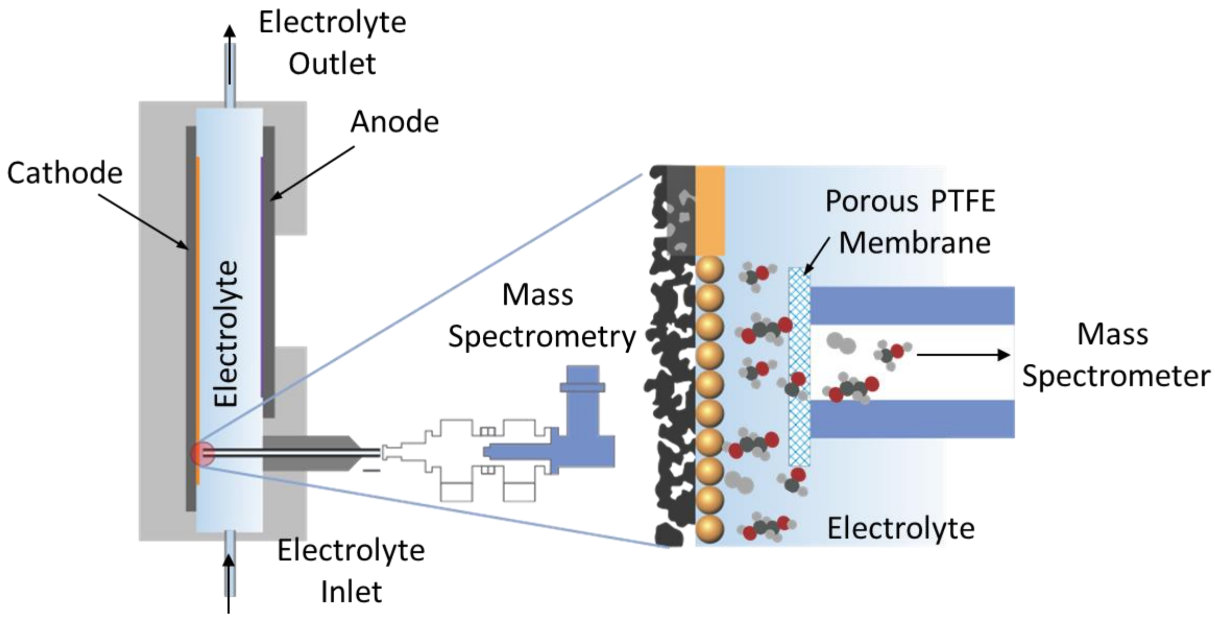

Figure S 18| Schematics of in-situ flow electrolyzer mass spectrometry (FEMS).

Supplementary note: DFT calculation

The graphene relaxation was calculated with a (6×6) supercell and 15 Å vacuum space, for which 3×3×1 Gamma-centered k-mesh was employed. The Cu (111) and Pd (111) surface relaxation was built by a (4×4) slab with 6 layers and at least 15 Å vacuum space, for which a 3×3×1 Gamma-centered k-point grid was employed.

Two reaction pathways are considered for C-C coupling process: (1) desorbed CH_2OH intermediates form ethylene glycol in the electrolyte, (2) two CH_2OH intermediates directly couple on the catalyst surface. In the first reaction pathway, the desorption of CH_2OH intermediates has a 0.62 eV thermodynamic barrier, and the kinetic barrier of the last step to form ethylene glycol is 0.28 eV as shown in Figure S 19. In the second reaction pathway, we applied the slow-growth approach[10,11] to calculate the structural change of the continuous process of direct coupling of two CH_2OH intermediates on the catalyst surface. In the slow growth calculations, the 300K temperature was kept by a Nose-Hoover thermostat and the $\partial\varepsilon$ was set to 0.0001 Å. The reaction coordinate in the slow growth is the distance between the carbon atoms in two CH_2OH intermediates, which decreases from 2.65 Å to 1.79 Å. Calculated results indicate that this process will eventually generate OH^- with a much higher kinetic barrier, rather than ethylene glycol. Therefore, the desorption of CH_2OH is more favorable followed by two CH_2OH intermediates coupling in the solution, suggesting that the desorption of CH_2OH intermediate is decisive in forming ethylene glycol on the surface of carbon catalyst.

The edge sites are also investigated due to their enrichment in π electrons that might function as the adsorption site. Two same pathways are considered on zigzag edge sites. The result of the slow-growth calculation shows similar C-C direct coupling behavior to that on the surface without ethylene glycol generated. At the same time, the adsorption energy of the CH_2OH intermediate on zigzag edges is up to 1.73 eV, which means there is less probability for the intermediates to desorb and be involved in the reaction. All of these confirm that C-C coupling and the formation of ethylene glycol base on the desorption of CH_2OH intermediate on catalyst surface.

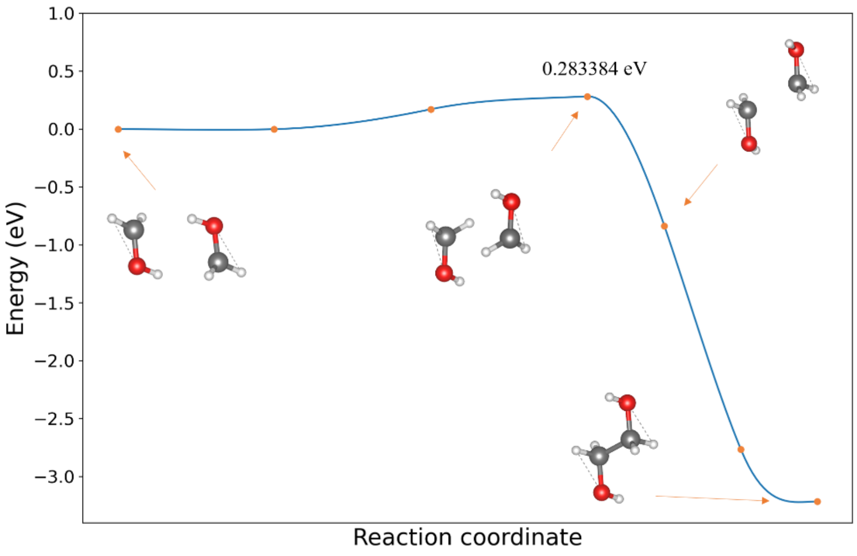

Figure S 19| Reaction path from two CH_2OH intermediates to ethylene glycol calculated by NEB approach. The illustration shows the structure of the initial state, final state and two transition states.

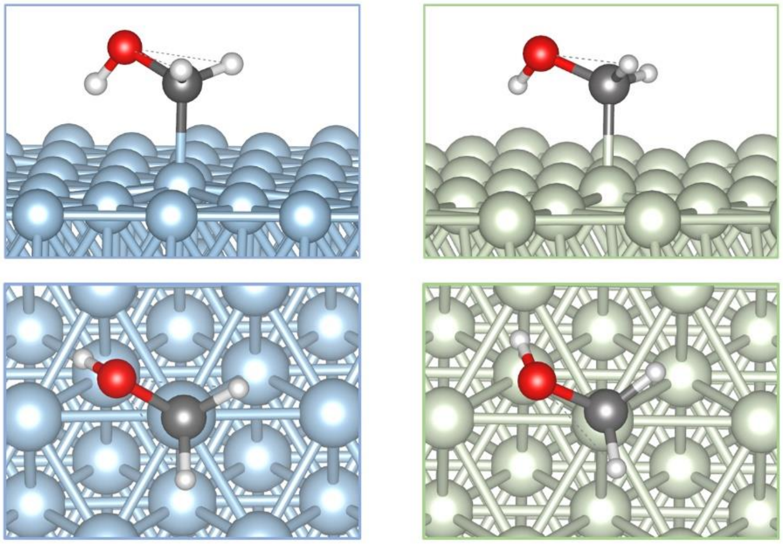

Figure S 20| The most favorable adsorption sites and configurations of CH_2OH intermediates on (1 1 1) surfaces of Cu and Pd.

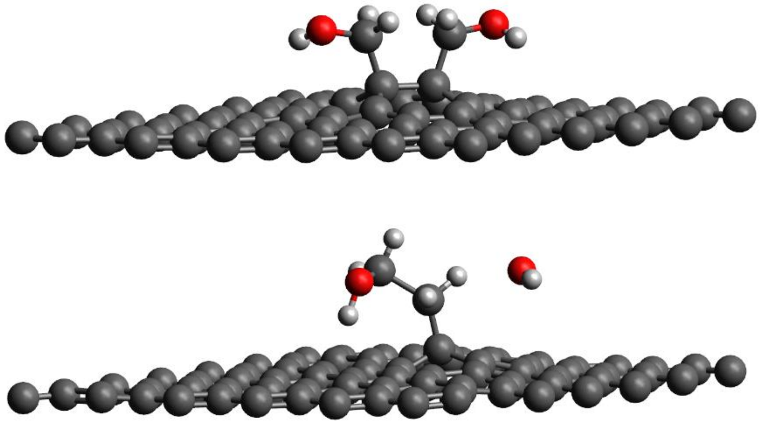

Figure S 21| The initial and final structures of two CH_2OH intermediates adsorbed on adjacent carbon atoms in the slow-growth calculation, which simulates the C-C direct coupling on the graphene surface.

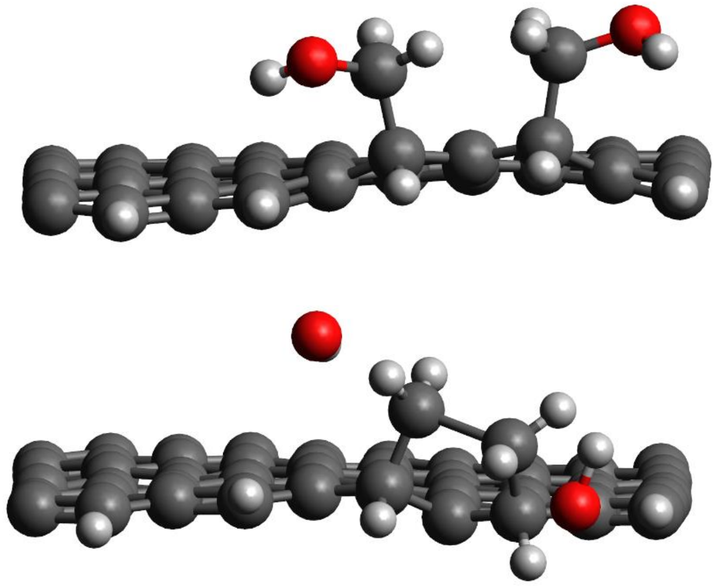

Figure S 22| The initial and final structures of two CH_2OH intermediates adsorbed on adjacent edge sites in the slow growth calculation, which simulates the C-C direct coupling on the zigzag edge sites.

Table S 2| Adjusted parameters from previously published electrochemical production model.[12]

Parameter	Value
Product production:	100000 kg/day
Product Selling Price:	0.838 $/kg
Operating time	350 days/year
Lifetime	20 years
Electricity Price	0.03 $/kWh
Current Density:	0.1 A/cm^2

Table S 3|Selectivity of electrochemical formaldehyde reduction on various catalysts at a current density of 25 mA cm^{-2}.

Catalysts	Hydrogen Faradaic efficiency / %	Ethylene glycol Faradaic efficiency / %	Methanol Faradaic efficiency / %
Co	84.3	0.0	15.7
Ni	73.2	0.0	26.8
Ag	4.1	0.0	95.9
Cu	0.0	0.0	100.0
Pd	12.7	0.0	87.3
C	3.4	47.0	49.6

Table S 4| Relative intensity of ^{12}C and ^{13}C in ethylene glycol produced from formaldehyde reduction using carbon catalysts. The ^{13}C-labeled formaldehyde ($^{13}CH_2O$) and non-labeled formaldehyde ($^{12}CH_2O$) were used as starting feedstock, respectively.

m/z / amu	Relative intensity	
	$^{12}CH_2O$	$^{13}CH_2O$
61	0.45	0.03
62	1.00	0.11
63	0.07	0.49
64	0.02	1.00

Table S 5| Ethylene glycol Faradaic efficiency on carbon black and oxygen-doped carbon black at various current density.

Current density / mA cm^{-2}	Ethylene glycol Faradaic efficiency / %		
	Carbon black	HNO_3 -carbon black	E-oxidized carbon black
10	47.0	41.6	11.0
25	60.0	30.0	15.9
50	47.5	18.7	16.7
100	29.3	11.4	6.1
200	16.2	7.4	4.7

Table S 6| Concentration dependence of formaldehyde electroreduction in 10 wt%, 20 wt%, 30 wt% and 37 wt% formaldehyde solution.

Formaldehyde concentration	Potential / V versus RHE	Ethylene glycol partial current density / mA cm^{-2}	Error bar
37%	-1.07	5.1	0.5
37%	-1.13	15.0	0.7
37%	-1.16	20.8	3.6
37%	-1.19	28.7	3.8
30%	-1.09	6.0	0.1
30%	-1.13	12.5	1.5
30%	-1.18	23.9	4.2
30%	-1.22	34.3	3.8
20%	-1.09	4.5	0.3
20%	-1.13	10.2	0.9
20%	-1.16	16.7	1.7
20%	-1.21	27.3	5.0
20%	-1.22	32.9	4.8
10%	-1.09	2.3	0.7
10%	-1.14	5.9	2.0
10%	-1.17	11.6	2.4
10%	-1.21	20.8	4.3
10%	-1.23	30.7	4.1

Table S 7| Temperature dependence of formaldehyde reduction at 30°C, 40°C, 50°C, respectively.

Temperature / °C	Potential / V versus RHE	Ethylene glycol partial current density / mA cm^{-2}	Error bar
30	-1.07	5.1	0.5
30	-1.13	15.0	0.7
30	-1.16	20.8	0.6
30	-1.19	28.7	1.5
30	-1.24	29.3	3.3
30	-1.31	32.5	6.6
40	-1.02	7.8	0.9
40	-1.06	21.8	1.7
40	-1.09	32.1	2.0
40	-1.11	43.8	5.1
40	-1.15	50.6	3.5
40	-1.20	63.9	7.7
50	-0.95	8.8	1.3
50	-1.00	20.8	1.5
50	-1.04	49.5	3.0
50	-1.06	66.8	6.8
50	-1.08	84.7	5.9
50	-1.13	145.6	8.6

Table S 8| Performance of formaldehyde electroreduction on carbon under the optimal condition (37% formaldehyde solution containing 1 M sodium acetate as supporting electrolyte, 50°C)。

Potential / V versus RHE	Current density / mA cm^{-2}	Ethylene glycol Faradaic efficiency / %	Error bar	H_2 Faradaic efficiency / %	Error bar	Methanol Faradaic efficiency / %
-0.93	10	87.98	1.53	3.11	3.85	8.91
-1.00	25	83.22	2.43	2.31	6.15	14.46
-1.04	50	93.08	3.07	3.85	6.55	3.07
-1.06	75	89.06	2.98	3.29	3.55	7.65
-1.09	100	84.73	3.66	4.62	6.35	10.65
-1.11	200	72.78	4.17	9.80	3.65	17.42

Table S 9| Total current density versus applied potential for methanol partial oxidation reaction on Pt nanoparticles and Pt microparticles, respectively.

Catalyst	Potential/ V vs RHE	Error bar	Current density / mA cm^{-2}
Pt nanoparticles	1.28	0.06	5
Pt nanoparticles	1.47	0.02	10
Pt nanoparticles	1.65	0.04	20
Pt nanoparticles	1.74	0.01	30
Pt nanoparticles	1.80	0.03	40
Pt microparticles	1.40	0.02	5
Pt microparticles	1.58	0.02	10
Pt microparticles	1.72	0.03	20
Pt microparticles	1.84	0.02	30
Pt microparticles	1.92	0.04	40

Table S 10| Formaldehyde Faradaic efficiency for methanol partial oxidation reaction on Pt microparticles and Pt nanoparticles.

Catalyst	Current density / mA cm^{-2}	Formaldehyde Faradaic efficiency / %	Error bar
Pt microparticles	10	73.4	5.2
Pt microparticles	20	67.0	4.1
Pt microparticles	30	70.4	4.2
Pt microparticles	40	55.5	3.0
Pt microparticles	50	51.4	6.2
Pt nanoparticles	10	27.4	7.4
Pt nanoparticles	20	21.2	3.6
Pt nanoparticles	30	17.1	4.5
Pt nanoparticles	40	14.9	3.8
Pt nanoparticles	50	14.8	3.4

Table S 11| Cell voltage and internal resistance of formaldehyde electroreduction reaction in different cell configurations.

Electrolyzer configuration	Cell voltage / V	Resistance / Ω
Two compartment flow electrolyzer	7.2	5.4
Membrane electrode assembly electrolyzer	3.2	0.7

Table S 12| Dependence of total cost of ethylene glycol production from methanol on operating cell voltage.

Cell voltage / V	Total cost / USD ton^{-1}
2	610.5
3.2	656.7
5.2	733.7
7.2	810.7
10	918.5

Table S 13| Performance of ethylene glycol electrosynthesis via coupling anodic methanol partial oxidation with formaldehyde electroreduction in a single membrane-electrode-assembly electrolyzer.

Current density / mA cm^{-2}	Cell voltage / V	Ethylene glycol Faradaic efficiency / %	Formaldehyde Faradaic efficiency / %
25	1.92	18.4	73.4
50	2.24	57.1	58.5
100	3.22	75.7	50.4
200	3.59	75.4	40.2

Supplementary References:

1 "Methanol price." *Trading Economics*, Feb 10, 2023, https://tradingeconomics.com/commodity/methanol

2 "Formaldehyde price trend and forecast." *Chemanalyst*, Feb 10, 2023, https://www.chemanalyst.com/Pricing-data/formaldehyde-1214

3 "Price of ethylene worldwide from 2017 to 2022." *Statista*, Feb 10, 2023, https://www.statista.com/statistics/1170573/price-ethylene-forecast-globally

4 "Ethylene Glycol Price Trend." *Procurement resource*, Feb 10, 2023, https://www.procurementresource.com/resource-center/ethylene-glycol-price-trends

5 Kim, J.-H., Kim, S.-H., Kim, B.-J. & Lee, H.-M. Effects of Oxygen-Containing Functional Groups on the Electrochemical Performance of Activated Carbon for EDLCs. *Nanomaterials* **13**, 262, (2023).

6 Intan, N. N. & Pfaendtner, J. Composition of Oxygen Functional Groups on Graphite Surfaces. *The Journal of Physical Chemistry C* **126**, 10653-10667, (2022).

7 Winkelman, J., Voorwinde, O., Ottens, M., Beenackers, A. & Janssen, L. Kinetics and chemical equilibrium of the hydration of formaldehyde. *Chemical Engineering Science* **57**, 4067-4076, (2002).

8 Jouny, M., Luc, W. & Jiao, F. High-rate electroreduction of carbon monoxide to multi-carbon products. *Nature Catalysis* **1**, 748-755, (2018).

9 Liu, X. *et al.* Henry's Law Constant and Overall Mass Transfer Coefficient for Formaldehyde Emission from Small Water Pools under Simulated Indoor Environmental Conditions. *Environmental Science & Technology* **49**, 1603-1610, (2015).

10 Woo, T. K., Margl, P. M., Blöchl, P. E. & Ziegler, T. A Combined Car−Parrinello QM/MM Implementation for ab Initio Molecular Dynamics Simulations of Extended Systems: Application to Transition Metal Catalysis. *The Journal of Physical Chemistry B* **101**, 7877-7880, (1997).

11 Zhao, X. & Liu, Y. Unveiling the Active Structure of Single Nickel Atom Catalysis: Critical Roles of Charge Capacity and Hydrogen Bonding. *Journal of the American Chemical Society* **142**, 5773-5777, (2020).

12 Jouny, M., Luc, W. & Jiao, F. General Techno-Economic Analysis of CO_2 Electrolysis Systems. *Industrial & Engineering Chemistry Research* **57**, 2165-2177, (2018).
